# Sweet Syndrome in a Patient With Acute Myeloid Leukemia: A Diagnostic Challenge in the Setting of Chemotherapy-Induced Neutropenia

**DOI:** 10.7759/cureus.110264

**Published:** 2026-06-04

**Authors:** Azza Abdalla, Jasdeep S Bajwa

**Affiliations:** 1 Internal Medicine, Rochester General Hospital, Rochester, USA

**Keywords:** acute myeloid leukemia (aml), fever of unknown origin (fuo), fever with rash, malignancy-associated sweet syndrome, neutrophilic dermatoses

## Abstract

A 69-year-old male with acute myeloid leukemia (AML) undergoing chemotherapy developed erythematous skin lesions at sites of prior intravenous access, along with fever and elevated inflammatory markers. Initially suspected of having resistant cellulitis or disseminated infection, his condition did not improve despite broad-spectrum antibiotics. Skin biopsy confirmed Sweet syndrome, and the patient showed rapid clinical improvement following corticosteroid therapy, with resolution of fever and skin lesions within 72 hours. This case underscores the importance of considering Sweet syndrome in neutropenic patients with unexplained fever and skin lesions, as early recognition and treatment can prevent unnecessary antibiotic use and ensure timely continuation of chemotherapy.

## Introduction

Sweet syndrome (SS), or acute febrile neutrophilic dermatosis, is a rare inflammatory condition first described by Robert Douglas Sweet in 1964 [1]. It is considered one of the neutrophilic dermatoses, which include pyoderma gangrenosum and subcorneal pustular dermatosis. It is characterized by painful, erythematous, and edematous skin lesions, often accompanied by systemic symptoms such as fever, malaise, and leukocytosis [2].

The diagnostic criteria for SS were first described in 1986 by Su and Liu [3] and were revised in 1994 by von den Driesch [4]. As defined by the latter, there are two major criteria and four minor criteria for SS. A patient is diagnosed with SS if both major criteria and at least two minor criteria are met. The major criteria consist of: (1) abrupt onset of tender or painful erythematous plaques or nodules, occasionally with vesicles, pustules, or bullae; and (2) predominantly neutrophilic infiltration in the dermis without leukocytoclastic vasculitis. The minor criteria include: (1) skin eruption preceded by a nonspecific respiratory or gastrointestinal tract infection or vaccination, or associated with inflammatory diseases such as chronic autoimmune disorders and infections, hemoproliferative disorders or solid malignant tumors, and pregnancy; (2) periods of general malaise and fever (>38°C); (3) laboratory values at onset including ESR >20 mm/hour, elevated C-reactive protein, positive segmented neutrophils and band forms >70% on peripheral blood smear, and leukocytosis >8000/mm³ (three of these four findings are required); and (4) excellent response to treatment with systemic corticosteroids or potassium iodide.

SS commonly affects females, with a female-to-male ratio of approximately 2-3:1 [5]. It can occur at any age but most commonly affects women between 30 and 50 years of age, whereas in men it tends to present later, typically between 50 and 90 years of age [5]. It is classified into three major subtypes: classic or idiopathic (mostly associated with infections and autoimmune disease), malignancy-associated or paraneoplastic, and drug-induced [6].

About 38%-53% of cases are classified as classic or idiopathic [7,8]. Drug-induced SS represents approximately 4%-24% of cases [7,8]. Among the subtypes, malignancy-associated SS holds particular clinical significance because of its frequent association with hematologic malignancies, most notably acute myeloid leukemia (AML). It was first described by Cohen and Kurzrock and accounts for approximately 20% of SS cases [9]. The temporal association between SS and leukemia is variable; it may precede, coincide with, or follow the diagnosis of leukemia [9]. Importantly, the emergence of SS in previously treated leukemia patients may serve as a sentinel sign of relapse or progression, underlining its clinical importance [10].

The histopathological hallmark of SS is a dense dermal infiltrate of mature neutrophils without evidence of leukocytoclastic vasculitis [11]. The pathogenesis is not entirely understood but is thought to involve dysregulation of the innate immune system, particularly the recruitment and activation of neutrophils [7]. Cytokines such as granulocyte colony-stimulating factor (G-CSF), interleukin-1 beta (IL-1β), interleukin-6 (IL-6), and interleukin-17 (IL-17) have been implicated in the recruitment and activation of neutrophils in the dermis [12]. In malignancy-associated SS, the aberrant immune signaling pathways of the underlying hematologic disease may exacerbate this inflammatory process [13].

Neutropenic fever occurs in patients with significantly reduced neutrophil counts and is often attributed to infection. Persistent fever in neutropenic patients may result from infectious or noninfectious causes. While prompt evaluation and empiric antibiotics are essential, a negative infectious workup should prompt consideration of alternative etiologies to avoid unnecessary antibiotic exposure. Important noninfectious causes include deep vein thrombosis (DVT)/pulmonary embolism (PE), hematoma, stroke, adrenal insufficiency, thyrotoxicosis, vasculitis, connective tissue diseases, chemotherapy-induced mucosal injury, and acute GVHD. SS should be considered an important differential diagnosis in neutropenic fever associated with skin lesions, particularly in patients with hematologic malignancies [14].

This case highlights the diagnostic challenges encountered in distinguishing SS from other causes of fever and cutaneous lesions in the setting of chemotherapy-induced neutropenia. The overlap in clinical presentation with infectious and other inflammatory conditions can complicate diagnosis and management, particularly in immunocompromised patients, where prompt recognition is essential to avoid unnecessary antimicrobial therapy and ensure appropriate treatment.

## Case presentation

A 69-year-old male with a past medical history of AML, hypertension, and hyperlipidemia presented to the hospital with generalized fatigue that had been worsening over two weeks and new-onset pain around the peripherally inserted central catheter (PICC) line (Mediport) used for chemotherapy.

The diagnosis of AML was made two months prior to presentation. Initial bone marrow biopsy showed 90% myeloblasts, with positive CD33 and CD34 on flow cytometry. Molecular testing showed a normal FISH panel and karyotype. It was also negative for NPM1 and FLT3 gene mutations. Next-generation sequencing revealed TET2 and ASXL1 gene mutations. Induction therapy with Dacogen/Venetoclax was started, and the patient completed the first cycle while admitted to the hospital.

**Figure 1:**
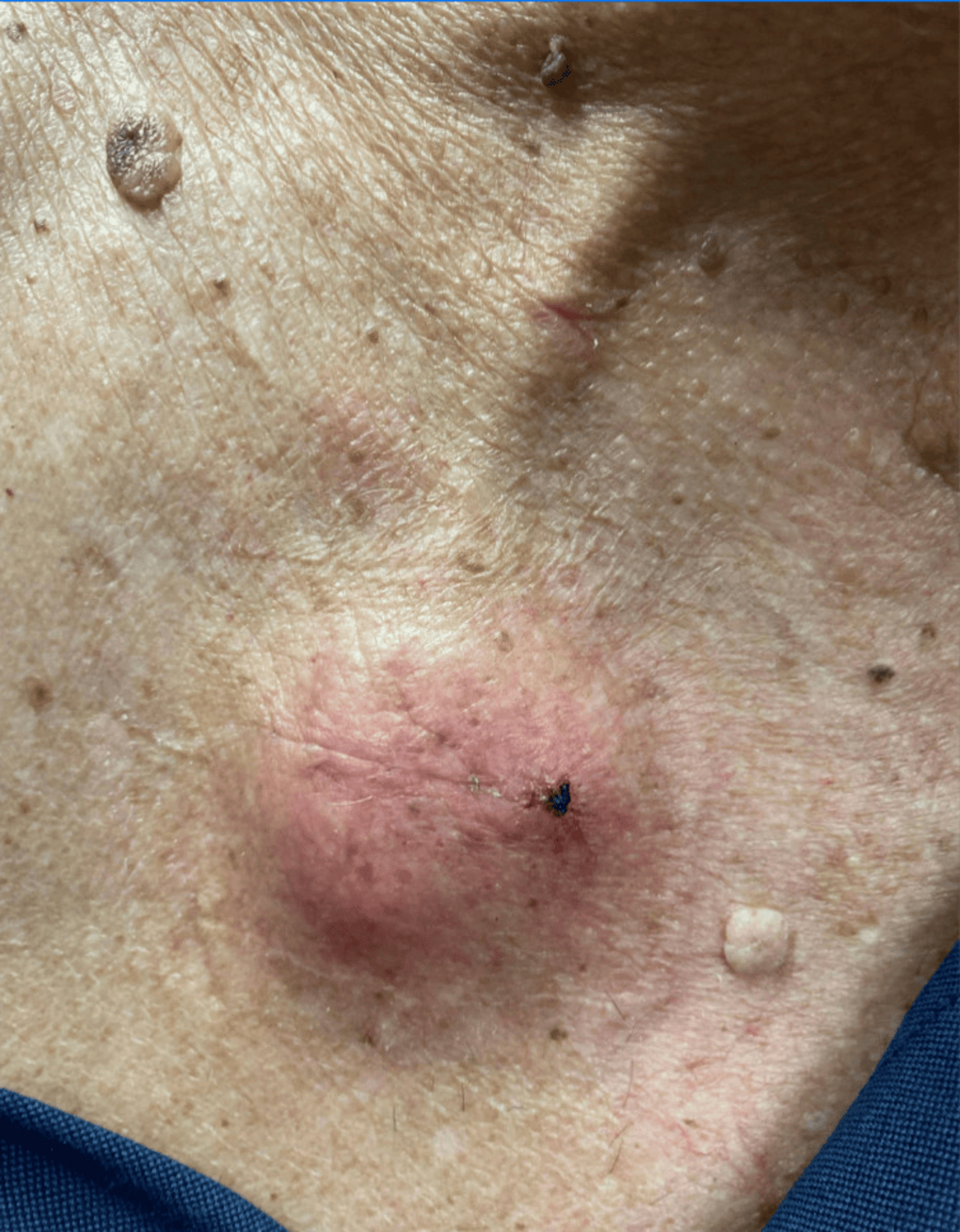
Initial skin lesion at the site of Mediport 2 x 2 cm "estimated" well-demarcated circular, slightly raised erythematous skin plaque at Mediport site.

The patient underwent PICC line insertion (Mediport) over his chest and was started on cycle 2 of chemotherapy in the outpatient setting. Five days later, he reported redness and tenderness near his Mediport. He reported no recent fevers or chills and denied any other symptoms. The patient was afebrile and vitally stable. Physical examination showed a circular, slightly raised erythematous skin patch at the Mediport site, as shown in Figure 1. The mucous membranes showed no mucositis, petechiae, or thrush. The neck was supple without lymphadenopathy. The remainder of the examination was unremarkable. He was then prescribed cephalexin, and the Mediport was removed. 

On day 25 of the second cycle of chemotherapy, the patient presented to the emergency department with complaints of near-syncope episodes and left jaw swelling with minimal pain. He denied fever and chills and reported no other symptoms. He was afebrile and vitally stable. On examination, the patient had a small area of erythema around the left ear, and the left anterior cheek was swollen. The Mediport site on the right chest was erythematous and edematous.

His workup is summarized in Table 1. Laboratory testing showed severe neutropenia with an absolute neutrophil count (ANC) of 0, anemia, and significantly elevated inflammatory markers. The chest X-ray was grossly unremarkable at the Mediport site. CT imaging of the mandible showed mild inflammatory stranding in the subcutaneous soft tissues overlying the body of the left mandible. No drainable abscess was identified.

**Table 1:** Summary of laboratory testing and reference values

Test	Result	Reference Range
White blood cell count (WBC)	0.6	4.0-10.8 ×10³/µL
Absolute neutrophilic count (ANC)	0	1.5-6.5 cell/ µL
Hemoglobin (Hb)	7.9	11.5-16.0 g/dL
Platelets	456	150-450 × 10³/µL
Lactate	1.1	0.5-2.2 mmol/L
C-reactive protein (CRP)	172	0.1-7.9 mg/dL
Erythrocyte sedimentation rate (ESR)	65	0-20 mm/hr
Creatinine	0.6	0.67-1.17 mg/dL
Aspartate aminotransferase (AST)	30	0-50 U/L
Alanine aminotransferase (ALT)	12	0-50 U/L

The patient was started on broad-spectrum antibiotics to cover both presumed Mediport site cellulitis and mandibular infection (cefepime, vancomycin, and Flagyl) after blood cultures and wound culture from the Mediport site were obtained. A dental consultation was performed, which suggested extraction of one periodontal tooth when the patient was medically stable in the outpatient setting. He also continued to receive posaconazole and acyclovir for prophylaxis. Chemotherapy was held on day 26 of the second cycle, and the patient was started on Granix (G-CSF).

**Figure 2:**
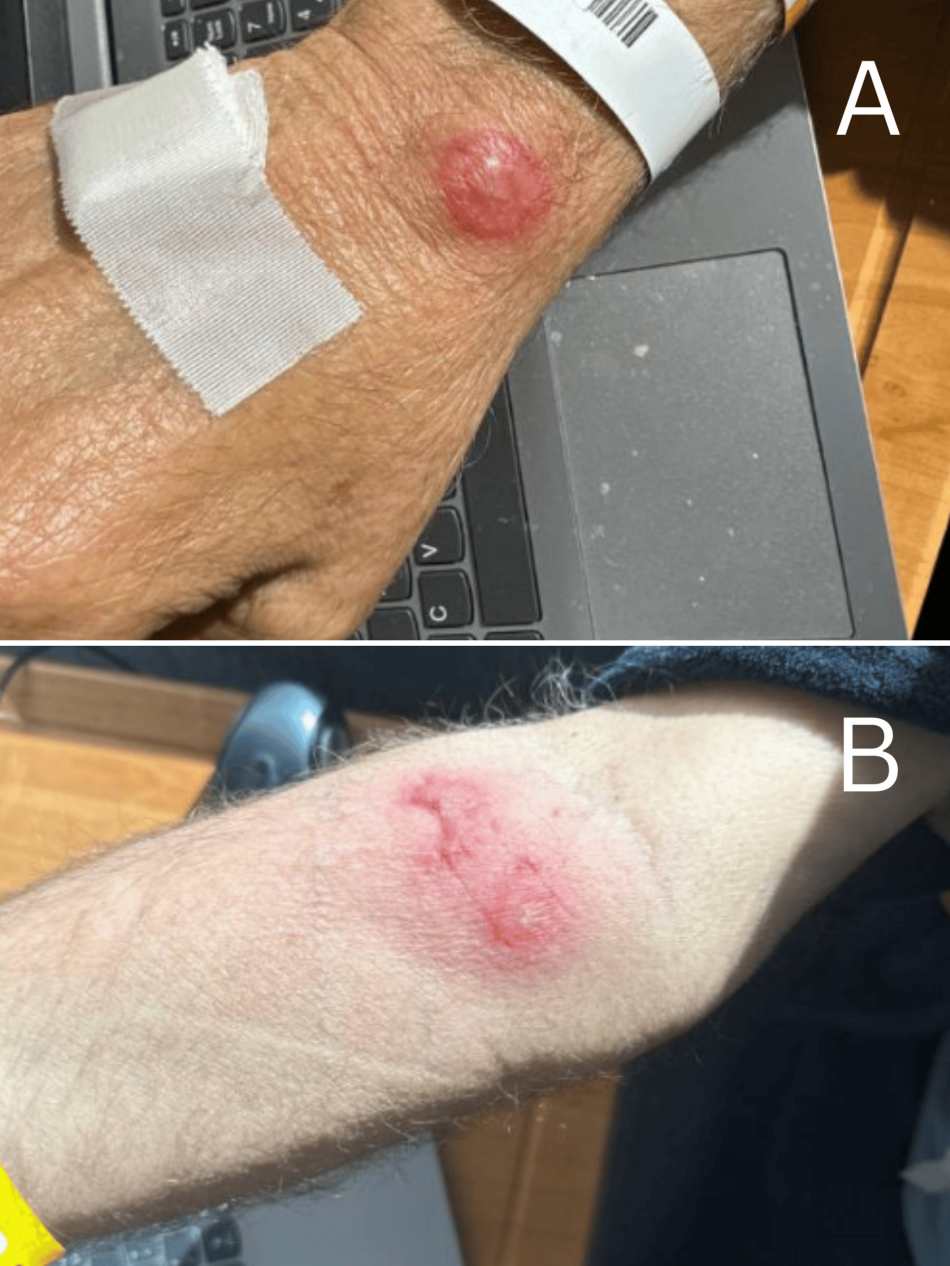
Progression of cutaneous lesions Bright red circular pseudovesicular papules at the dorsal surface of the right wrist (1.5 x 1.5 cm by estimation) (A) and right antecubital fossa (2 fused lesions) (B).

The patient started to develop fevers while hospitalized, with a temperature of 38.8°C (101.8°F), and blood cultures showed no growth. New bright red, circular, painful pseudovesicular papules also started to appear in the right antecubital fossa surrounding a peripheral IV access site and on the dorsal surface of the right wrist (Figure 2). At this point, resistant cellulitis versus disseminated fungal infection was considered, and a fungal workup was initiated. His ANC increased slightly to 0.4 after initiation of G-CSF. 

At this point, a noninfectious etiology behind the symptoms was considered. Given the IV site reactions, one at the peripheral IV site and the other at the Mediport site, pathergy was considered, which is a classic finding of neutrophilic dermatoses. Differential diagnoses that were considered included cellulitis, given the fever and elevated inflammatory markers; pyoderma gangrenosum and Behçet disease, given the pathergy; and erythema nodosum, given the painful nodules.

Subsequently, Granix was held, and a skin biopsy was performed, which showed a marked neutrophilic dermal inflammatory infiltrate accompanied by extravasated red cells and fibrin. No vasculitis was identified. The findings were consistent with a neutrophilic dermatosis and compatible with SS.

**Figure 3:**
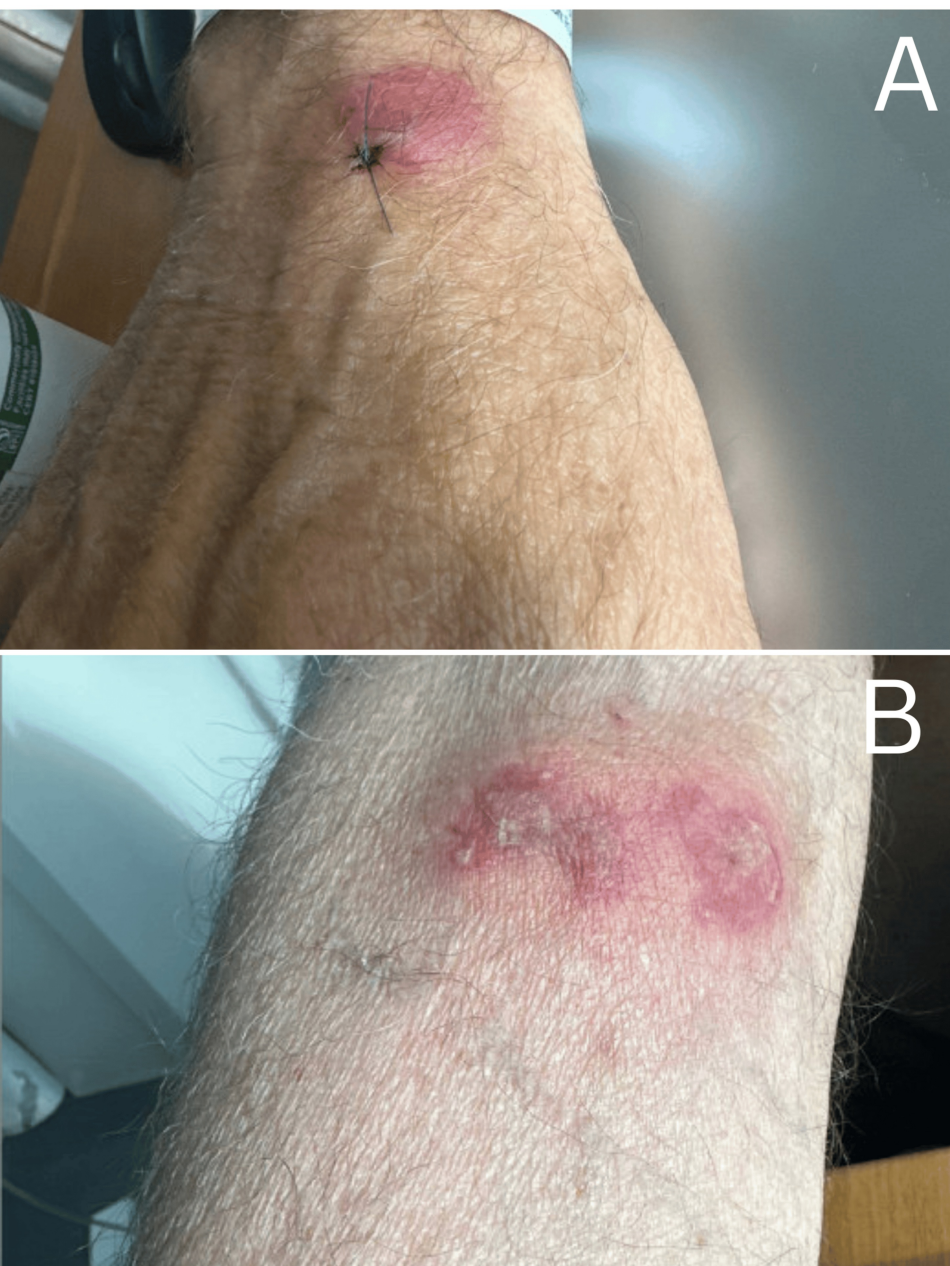
Improvement of the rash after 72 hours of initiation of steroids Figure shows improvement of lesions at dorsal surface of the right wrist (A) and right antecubital fossa (B)

High-dose IV steroids were started. After 24 hours of steroid initiation, the fever completely subsided. After 48 hours, the jaw swelling had significantly improved. Seventy-two hours later, improvement was noted in the erythema at the prior Mediport site on the right chest, as well as on the right wrist and antecubital fossa (Figure 3). Given this response, SS was confirmed, as the patient met all major and minor diagnostic criteria [4].

## Discussion

SS, or acute febrile neutrophilic dermatosis, is a rare but well-recognized complication in patients with hematologic malignancies, which account for 85% of cancer-related SS, most commonly AML [5]. It affects males and females equally, unlike other subtypes of SS, which affect females predominantly [7,15]. The incidence of SS in AML was 1% in a study that included 2178 patients with AML [16].

This case highlights the diagnostic challenges in managing a patient with SS occurring in the context of chemotherapy-induced neutropenia with multiple probable causes. Given the temporal appearance of the lesions, each was initially attributed to a different pathology, such as IV site infection causing cellulitis, a neck lesion related to tooth infection, and pathergy. A noninfectious etiology became more prominent after the appearance of the wrist lesion, which was unrelated to a clear source of infection. Although disseminated infections are often considered in febrile neutropenia, SS can resemble disseminated bacterial, fungal, and mycobacterial infections, especially nodular SS [11].

The paradoxical finding of a dense, diffuse neutrophilic infiltrate on histopathology despite a peripheral ANC of zero may be explained by local chemotactic gradients, often driven by cytokines and chemokines such as IL-8, TNF-α, or IL-17, which can sequester, arrest, and concentrate the remaining, newly released, or marginated neutrophils directly into the skin or target organs, thereby depleting the circulating pool. Peripheral neutropenia should never deter a clinician from performing a skin biopsy when a neutrophilic dermatosis is suspected [17].

Pathergy, a phenomenon in which minor trauma triggers inflammatory skin lesions, was suspected because the patient had lesions at the IV and Mediport sites. It is a shared manifestation that occurs in other neutrophilic dermatoses [11,18]. Given the absence of oral and genital ulcers and the morphological features of the rash, the diagnoses of Behçet disease and pyoderma gangrenosum (PG) became less likely, although flaccid bullous SS with subsequent ulceration can present with features similar to the rash seen in PG [11]. The distribution and nature of the skin lesions were more consistent with SS, which can involve the upper extremities, neck, and torso, all of which were affected in this patient [19].

The pathophysiology of SS is believed to involve an exaggerated neutrophilic response to various triggers, including infections, malignancies, and medications [13]. The importance of identifying the underlying cause of SS lies in improving patient outcomes, as it may decrease interruption of necessary management, including chemotherapy and other medications related to the underlying cancer, such as G-CSF and decitabine.

Decitabine and other chemotherapy agents contributing to SS have been described in case reports [20-22], with no longitudinal studies reported in the literature. However, one case report described the death of a patient due to failure to initiate chemotherapy after decitabine-induced SS [20].

G-CSF is considered the most common drug associated with drug-induced SS [18], as it can cause a vigorous neutrophilic response by inducing growth factors and cytokines. However, in this case, it was started after the appearance of the first two lesions at presentation. It is likely that G-CSF contributed to the progression of SS, with AML being the underlying cause. This is evident from the course of the disease, including the fever curve and progression of the lesions.

AML, a well-known trigger of SS, is linked to impaired neutrophil functions, including adhesion, migration, chemotaxis, and phagocytosis, which contribute to neutrophilic infiltration [23]. The fact that the patient was diagnosed with AML nearly six months before the onset of SS raises an interesting question: Was AML the primary triggering factor, or did it merely play a contributory role alongside other factors? Our patient had a normal karyotype and FISH (fluorescence in situ hybridization) panel and lacked FLT3 and NPM1 mutations, which have previously been reported in association with SS [16]. However, next-generation sequencing identified TET2 and ASXL1 mutations, both of which are involved in epigenetic regulation and have been linked to aberrant inflammatory signaling in myeloid malignancies [24,25]. While a direct causal relationship cannot be established, these findings support the possibility that the underlying leukemic clone contributed to the development of SS through dysregulated cytokine-mediated inflammation.

In this patient, high-dose intravenous steroids were initiated promptly, leading to significant clinical improvement within 24 to 72 hours, including resolution of fever, jaw swelling, and erythema at the Mediport site and peripheral IV sites. The clinical response to steroids supported the diagnosis, and antibiotics were discontinued once the patient was afebrile for 72 hours and no further signs of infection were evident.

## Conclusions

In patients with hematologic malignancies, SS (acute febrile neutrophilic dermatosis) should be considered in the differential diagnosis when neutropenic fever is accompanied by cutaneous eruptions in the absence of an identifiable infectious source. Because fever and skin lesions in immunocompromised patients are frequently presumed to be infectious, recognition of this entity can help guide appropriate diagnostic evaluation and management.

This case highlights the diagnostic challenge of distinguishing infectious from noninfectious causes of fever and skin lesions in patients with chemotherapy-induced neutropenia. Careful clinical assessment, exclusion of infection, and skin biopsy when feasible are essential for establishing the diagnosis and facilitating timely corticosteroid treatment, which in this patient resulted in rapid clinical improvement. Notably, the clinical course suggests that the underlying AML was the primary driver of SS, while G-CSF administration did not appear to contribute to disease exacerbation. Although broader benefits such as reduced antimicrobial exposure and shorter hospitalization have been reported in the literature, these outcomes cannot be directly inferred from this single case.
